# Population ageing and gastric cancer mortality burden in China, 2008–2021: a nationwide analysis with projections to 2036

**DOI:** 10.3389/fpubh.2026.1875254

**Published:** 2026-08-05

**Authors:** Haoyu Fang, Yuezhou Zhang, Yijiao Ning, Junhao Mu

**Affiliations:** 1 Department of Hepatobiliary Surgery, The Second Affiliated Hospital of Chongqing Medical University, Chongqing, China; 2 Department of Respiratory Medicine, The First Affiliated Hospital of Chongqing Medical University, Chongqing, China; 3 Department of Infectious Diseases, The First Affiliated Hospital of Chongqing Medical University, Chongqing, China

**Keywords:** age-period-cohort analysis, China, gastric cancer, population ageing, premature mortality burden

## Abstract

**Background:**

Gastric cancer remains a major cause of mortality in China, yet updated nationwide evidence integrating mortality trends, premature mortality burden, and future projections is limited. Using data from China’s Disease Surveillance Points (DSP) system, we evaluated long-term trends in gastric cancer mortality and demographic disparities.

**Methods:**

Gastric cancer deaths (ICD-10 code C16) from 2008 to 2021 were obtained from the national DSP system. Crude mortality rate (CMR), age-standardized mortality rate (ASMR), years of potential life lost (PYLL), and potential years of life lost rate (PYLLR) were estimated overall and by sex, residence (urban/rural), and region (eastern/central/western China). ASMRs were standardized to the 2010 Chinese standard population. Temporal trends were assessed using Joinpoint regression, age-period-cohort patterns using the National Cancer Institute age-period-cohort tool, and future ASMRs through 2036 using a Bayesian age-period-cohort model.

**Results:**

From 2008 to 2021, gastric cancer mortality declined substantially in China. CMR decreased from 20.64 to 18.39 per 100,000, while ASMR declined from 22.44 to 12.29 per 100,000. PYLLR also decreased from 1.38‰ to 0.83‰. Males and rural residents consistently had higher ASMRs and PYLLRs than females and urban residents. Regional disparities persisted, with ASMRs generally higher in eastern and central China than in western China, although the western region showed the steepest relative decline. Age-period-cohort analysis demonstrated a strong age effect, with mortality increasing sharply after age 60 and declining period and cohort effects over time. The burden of gastric cancer became increasingly concentrated among older adults. Model-based projections suggested continued declines in ASMR through 2036, although uncertainty widened over longer projection periods.

**Conclusion:**

Gastric cancer mortality and premature mortality burden declined substantially in China between 2008 and 2021, but important demographic and regional disparities remained. The larger decline in ASMR than in CMR, together with the increasing concentration of deaths among older adults, suggests that population ageing continues to reshape the future burden of gastric cancer.

## 1 Introduction

Gastric cancer remains a major cause of cancer-related death worldwide and continues to impose a substantial public health burden in China (1). As a high-burden country accounting for a large share of global gastric cancer cases and deaths, China is of particular importance for understanding contemporary mortality patterns and informing prevention strategies (2). Although gastric cancer control has improved in recent decades, the burden of mortality in China remains shaped by population ageing, demographic disparities, and regional heterogeneity (3, 4).

In countries undergoing rapid demographic transition, however, temporal changes in gastric cancer mortality cannot be fully captured by a single summary indicator. Age-standardized mortality rates (ASMR) reflect changes in underlying age-adjusted risk, whereas crude mortality rates (CMR) remain influenced by shifts in population age structure. In addition, measures of premature mortality, such as potential years of life lost (PYLL) and potential years of life lost rate (PYLLR), provide complementary information on the societal impact of gastric cancer beyond conventional mortality estimates.

Although several studies have described the burden of gastric cancer in China, important gaps remain. Many previous analyses were based on modelled estimates, ended before more recent years, or focused primarily on a single mortality indicator (5, 6). National surveillance-based evidence that jointly evaluates CMR, ASMR, PYLL, PYLLR, age-period-cohort patterns, and future trends remains limited. In particular, under conditions of rapid population ageing and ongoing changes in China’s mortality surveillance system, an updated assessment that considers both changes in mortality risk and shifts in burden distribution is needed.

Using data from China’s Disease Surveillance Points (DSP) system for 2008–2021, we aimed to assess temporal trends in gastric cancer mortality and premature mortality burden in mainland China overall and by sex, residence, and region. Specifically, we examined CMR, ASMR, PYLL, PYLLR, age-period-cohort patterns, and projected future mortality trends to provide an updated surveillance-based assessment of gastric cancer burden in the context of demographic change.

## 2 Materials and methods

### 2.1 Data source and study population

Gastric cancer deaths were identified using the International Classification of Diseases, 10th Revision (ICD-10) code C16. Mortality data for 2008–2021 were obtained from China’s DSP system, a nationwide mortality surveillance system administered by the Chinese Center for Disease Control and Prevention. We selected 2008 as the starting year because it was the earliest year for online registration, while 2021 was the most recent year available at the time of study design (7, 8).

The DSP system covered 161 surveillance points during 2008–2012 and was expanded to 605 points from 2013 onward, substantially increasing population coverage. To improve data quality, surveillance points with poor data completeness or implausibly low reported mortality were excluded according to the original data source criteria, and 155 points for 2008–2012 and 432 points for 2013–2021 were retained for analysis (9). Because the 2013 system expansion may affect the comparability of absolute death counts over time, this issue was taken into account when interpreting temporal changes, particularly for crude death numbers.

The DSP data used in the study were de-identified aggregated monitoring data, containing no personally identifiable information, and processed in accordance with the ethical principles of the Declaration of Helsinki (2013 edition).

### 2.2 Stratification variables

Analyses were stratified by sex (male/female), place of residence (urban/rural), and geographic region (eastern, central, and western China) according to the classification of the National Bureau of Statistics of China. In the DSP system, county-level units (including county-level cities) were classified as rural areas, whereas districts were classified as urban areas. Age was grouped as <1 year, 1–4 years, and subsequent 5-year intervals up to 85 years and older for mortality estimation and descriptive analyses.

### 2.3 Data processing and calculation

We calculated the CMR, ASMR, PYLL, and PYLLR. The CMR was defined as the number of gastric cancer deaths divided by the corresponding population and was expressed per 100,000 population. The ASMR was estimated by direct standardization using the 2010 Chinese standard population from the Sixth National Population Census. ASMRs were weighted by the corresponding age-specific proportions of the standard population and summed to obtain the standardized rate, expressed per 100,000 population.

Premature mortality burden was assessed using PYLL and PYLLR. PYLL was calculated as the sum of age-group-specific deaths multiplied by the remaining years of life below the selected premature mortality threshold: PYLL = *Σ* (ai * di), where di denotes the number of gastric cancer deaths in age group i and ai denotes the remaining years of life assigned to that age group. In the main analysis, 70 years was used as the upper age limit for premature mortality, and deaths occurring at ages 70 years and older did not contribute to PYLL (10). PYLLR was calculated by dividing PYLL by the corresponding population and was expressed per 1,000 population.

### 2.4 Statistical analysis

Temporal trends in ASMR and PYLLR were assessed using the Joinpoint Regression Program (version 5.4.0.0; National Cancer Institute, Bethesda, MD, USA). Joinpoint models were fitted using grid search and Monte Carlo permutation tests, with a maximum Joinpoint count of 4 (11, 12). Annual percentage change (APC) for each stage and the mean annual percentage change (AAPC) over the entire study period were estimated, along with 95% confidence intervals (CI).

The NCI age-period-cohort analysis tool^1^ was used to assess the impact of age, period, and birth cohort on gastric cancer mortality. The analysis was conducted using the National Cancer Institute APC Web Tool, which implements estimable APC functions for incidence and mortality rate data within a log-linear Poisson framework (7). Age-specific death counts and corresponding population denominators were arranged by 5-year age groups and calendar periods. Because gastric cancer mortality was negligible at younger ages, age-period-cohort analyses were restricted to individuals aged 20 years and older to improve interpretability and model stability. The APC model was used to estimate the net drift, local drift, longitudinal age curve, period rate ratios (RRs), and cohort RRs. Net drift represents the overall annual percentage change in mortality adjusted for age, period, and cohort effects, whereas local drift represents the age-specific annual percentage change. Period and cohort RRs were interpreted relative to the reference period and reference birth cohort specified by the APC Web Tool. In this study, the reference age, period, and birth cohort were 52.5 years, 2015.5, and 1963, respectively. Wald tests were used to assess the statistical significance of period and cohort effects.

Future ASMR trends from 2022 to 2036 were projected using a Bayesian age-period-cohort (BAPC) model implemented in R with the “BAPC” and “INLA” packages (13). Age-specific deaths and population denominators were arranged in five-year age groups, and the APC data object was constructed with a five-year grouping factor. The model was fitted separately for the total population, males, and females. In the BAPC framework, age, period, and cohort effects were assigned second-order random-walk smoothing priors, and an independent identically distributed random effect was included to account for extra-Poisson variation. Precision parameters were assigned the default log-gamma hyperpriors implemented in the BAPC/INLA framework. Projected population denominators for 2022–2036 were obtained from the Institute for Health Metrics and Evaluation population projections for China and were matched to the observed age groups; sex-specific projections used the corresponding sex-specific population projections (14). Predicted age-specific mortality rates were standardized to the 2010 Chinese standard population to derive projected ASMRs, with 95% credible intervals. The projection horizon was set to 2036 to provide a 15-year forecast beyond the most recent observed year (2021). To evaluate short-term predictive performance, we performed a back-testing analysis. The BAPC model was refitted using data from 2008 to 2017 only, with deaths in 2018–2021 treated as unobserved while the corresponding population denominators were retained. Predicted ASMRs for 2018–2021 were then compared with observed ASMRs using the mean absolute error (MAE), mean absolute percentage error (MAPE), and whether the observed ASMR fell within the 95% credible interval.

All statistical tests were two-sided, and a *p*-value <0.05 was considered statistically significant when applicable (15).

### 2.5 Sensitivity considerations

To assess the robustness of the main findings in relation to the 2013 expansion of the DSP system, we conducted a restricted-period sensitivity analysis for 2013–2021, representing the post-expansion phase with broader population coverage and more stable surveillance. Temporal trends in ASMR and PYLLR during 2013–2021 were evaluated using Joinpoint regression with no joinpoints allowed, given the limited number of annual observations within the restricted period. Under this specification, the estimated APC represented the average annual change during the post-expansion period.

To minimize the influence of changes in DSP coverage on absolute mortality counts, we estimated national deaths using a rate-based post-stratification approach rather than directly interpreting raw DSP death counts as national counts in the sensitivity analysis. For each calendar year, age- and sex-specific gastric cancer mortality rates were first calculated from the DSP data. These stratum-specific rates were then multiplied by the corresponding national age- and sex-specific population denominators, and estimated deaths were summed across strata to obtain national estimates overall and by sex. Age groups were harmonized to 0–4, 5–9, …, and ≥85 years for rate-based standardization and projection; the original <1 and 1–4 years groups in the DSP mortality table were combined into 0–4 years to match the national population denominators.

In response to concerns regarding the choice of the premature mortality threshold, we conducted an additional sensitivity analysis using 75 years as the upper age limit for PYLL. In this analysis, PYLL was recalculated as the sum of age-group-specific deaths multiplied by the remaining years of life below 75 years, using the formula PYLL = *Σ* (ai * di), where di denotes the number of deaths in age group i and ai denotes the remaining years of life. The remaining years were calculated as 75 - (mean age + 0.5) for each age group. The 0-year age group was treated as 0–1 years, the 1-year age group as 1–5 years, and subsequent age groups as five-year intervals; deaths at ages 75 years and older did not contribute to PYLL. PYLLR was recalculated accordingly, and temporal trends were reassessed using Joinpoint regression.

## 3 Results

### 3.1 Region patterns of gastric cancer ASMR and PYLLR in mainland China

**Figure 1:**
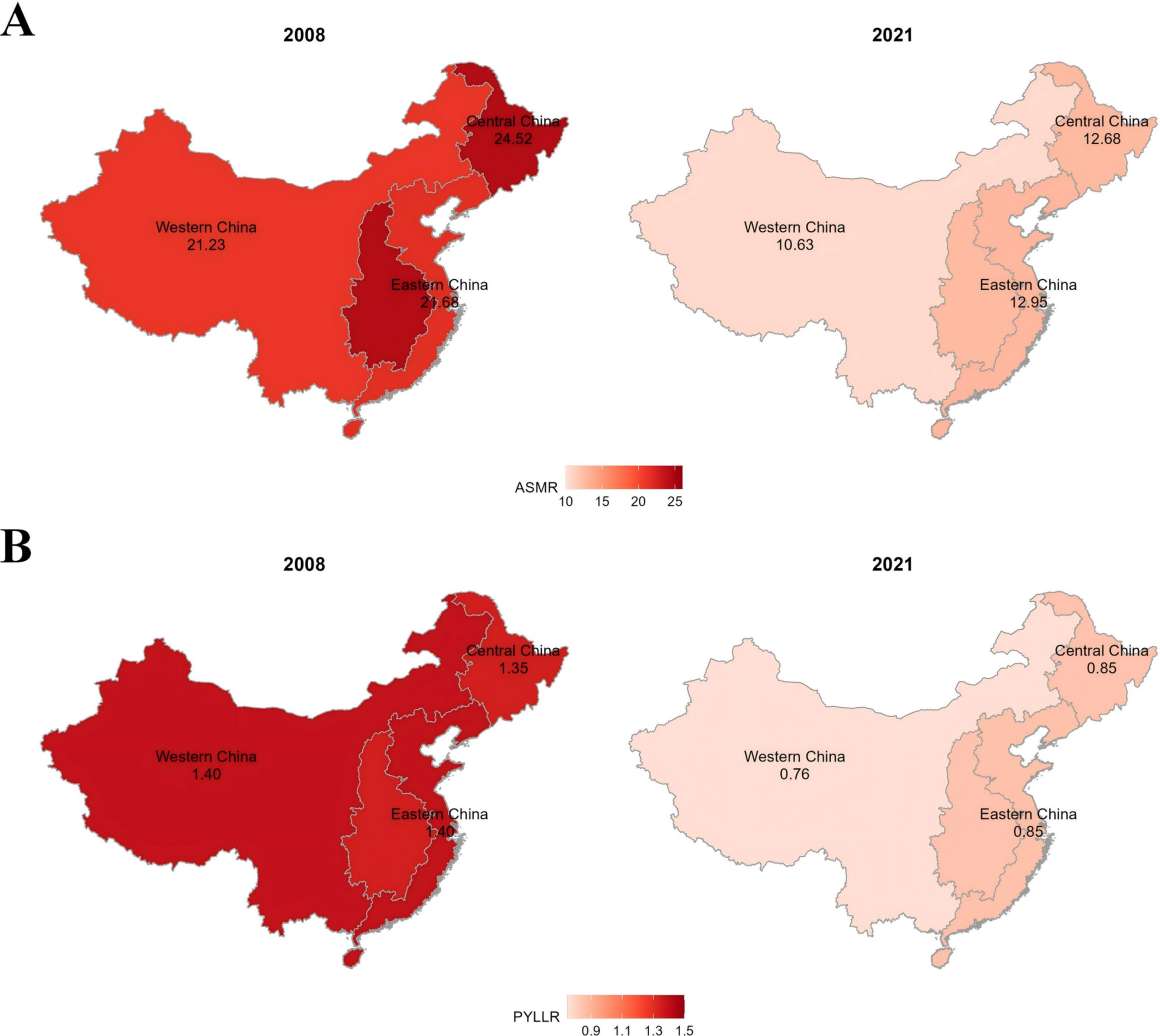
Spatial distribution of gastric cancer ASMR and PYLLR in mainland China, 2008 vs 2021. **(A)** Regional distribution of ASMR for gastric cancer in 2008 and 2021. **(B)** Regional distribution of years of PYLLR for gastric cancer in 2008 and 2021.

Figure 1 shows the spatial distribution of ASMR and PYLLR in gastric cancer in mainland China in 2008 and 2021. Darker colors represent higher ASMR and PYLLR. Overall, compared to 2008, ASMR and PYLLR in gastric cancer significantly decreased in eastern, central, and western China in 2021. Figure 1A shows the spatial distribution of ASMR in gastric cancer in China. In the first and last 2 years of our inclusion period, ASMR in Eastern China and Central China was higher than in Western China, indicating a regional imbalance in the risk of death from gastric cancer. Figure 1B shows the spatial distribution of PYLLR in gastric cancer in China in 2008 and 2021. Unlike ASMR, the regional differences in PYLLR appear to be insignificant. The PYLLR value was 1.40 in both Eastern China and Western China in 2008, and 0.85 in both Eastern China and Central China in 2021. We consider this to be related to the average age of death from gastric cancer being close to life expectancy.

### 3.2 Gastric cancer mortality rate and changing trends

**Figure 2:**
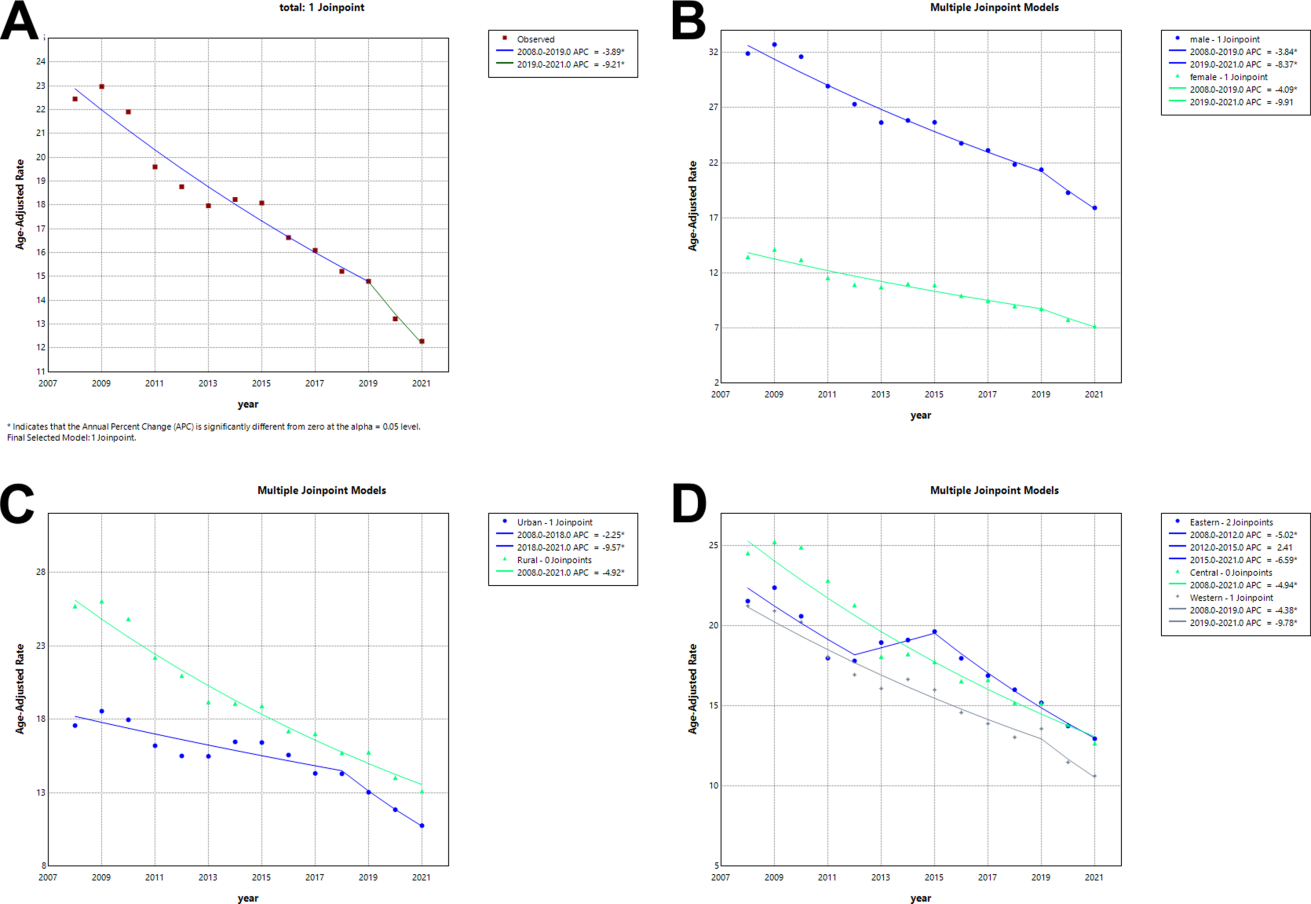
Joinpoint regression of gastric cancer ASMR trends in China, 2008–2021. Joinpoint regression analyses of ASMR for gastric cancer from 2008 to 2021. **(A)** Overall population. **(B)** By sex (male and female). **(C)** By residence (urban and rural). **(D)** By region (Eastern, Central, and Western China). APC for each segment is displayed in the legend; an asterisk indicates APC significantly different from zero at *α* = 0.05.

Between 2008 and 2021, the mortality rate from gastric cancer in China showed a significant downward trend. The CMR decreased from 20.64 cases per 100,000 people in 2008 to 18.39 cases per 100,000 people in 2021. The ASMR decreased even more significantly, from 22.44 cases per 100,000 people to 12.29 cases per 100,000 people. Joinpoint regression analysis confirmed the significant downward trend in ASMR, with an overall AAPC of −4.73% (95% CI: −5.90% to −3.54%; *p* < 0.01) (Table 1; Figure 2; Supplementary Figure 1).

**Table 1:** CMR and ASMR of gastric cancer from 2008 to 2021 in China (1/100,000).

Index	Measure	2008	2009	2010	2011	2012	2013	2014	2015	2016	2017	2018	2019	2020	2021	AAPC% (95%CI)	t	*p*
Male	CMR	27.83	28.34	27.54	26.56	27.28	27.61	28.39	28.24	27.72	27.09	26.74	26.65	25.11	25.10			
	ASMR	31.90	32.72	31.61	28.93	27.31	25.66	25.85	25.68	23.77	23.13	21.85	21.53	19.28	17.91	−4.55 (−5.60, −3.49)	−8.23	<0.01
Female	CMR	13.13	13.83	12.93	12.36	12.46	12.93	13.52	13.37	13.04	12.52	12.38	12.26	11.47	11.46			
	ASMR	13.44	14.12	13.18	11.54	10.92	10.72	11.00	10.91	9.92	9.49	8.98	8.83	7.75	7.16	−5.01 (−6.56, −3.43)	−6.11	<0.01
	X^2^	2376.97	1857.63	2061.47	1992.24	2119.57	6000.05	6646.87	6808.49	6947.45	7220.04	7130.78	7326.21	7028.62	6776.53			
	P	<0.01	<0.01	<0.01	<0.01	<0.01	<0.01	<0.01	<0.01	<0.01	<0.01	<0.01	<0.01	<0.01	<0.01			
Urban areas	CMR	18.55	19.63	18.57	16.14	16.72	18.52	19.19	19.24	18.82	17.45	18.32	17.68	16.96	16.27			
	ASMR	17.56	18.54	17.95	16.18	15.49	15.48	16.46	16.42	15.56	14.31	14.29	13.13	11.84	10.76	−3.99 (−5.35, −2.61)	−5.58	<0.01
Rural areas	CMR	21.77	22.12	21.49	21.86	22.19	21.26	22.00	21.72	21.37	21.18	20.40	20.55	19.18	19.49			
	ASMR	25.69	26.02	24.81	22.18	20.95	19.18	19.08	18.91	17.20	17.02	15.72	15.87	14.02	13.10	−4.92 (−5.48, −4.36)	−18.79	<0.01
	X^2^	84.41	49.99	77.71	310.00	277.10	178.66	207.12	164.61	187.81	423.18	133.95	261.97	170.51	340.62			
	P	<0.01	<0.01	<0.01	<0.01	<0.01	<0.01	<0.01	<0.01	<0.01	<0.01	<0.01	<0.01	<0.01	<0.01			
Eastern China	CMR	22.24	22.92	21.08	19.87	20.63	23.72	24.14	24.79	23.99	22.86	22.71	21.88	20.91	20.76			
	ASMR	21.68	22.36	20.58	17.97	17.80	18.96	19.12	19.65	17.98	16.89	16.01	15.31	13.75	12.95	−4.10 (−5.91, −2.25)	−4.30	<0.01
Central China	CMR	21.07	22.11	22.21	21.66	21.77	19.14	19.91	19.41	19.47	19.80	18.97	19.36	18.53	18.70			
	ASMR	24.52	25.22	24.89	22.80	21.27	18.08	18.24	17.75	16.55	16.64	15.20	15.29	13.83	12.68	−4.94 (−5.62, −2.26)	−15.47	<0.01
Western China	CMR	17.87	17.76	17.10	16.36	16.72	17.16	18.05	17.36	16.80	15.56	16.06	16.37	14.51	14.43			
	ASMR	21.23	20.91	20.21	18.04	16.93	16.09	16.66	16.00	14.59	13.89	13.84	13.68	11.48	10.63	−5.23 (−6.53, −3.91)	−7.60	<0.01
	X^2^	112.87	163.61	163.44	167.86	156.36	832.46	779.22	1227.31	1119.17	1127.51	993.05	680.99	982.97	936.87			
	P	<0.01	<0.01	<0.01	<0.01	<0.01	<0.01	<0.01	<0.01	<0.01	<0.01	<0.01	<0.01	<0.01	<0.01			
Total	CMR	20.64	21.24	20.39	19.58	20.01	20.41	21.10	20.92	20.51	19.92	19.69	19.57	18.40	18.39			
	ASMR	22.44	22.96	21.89	19.59	18.76	17.98	18.24	18.09	16.64	16.10	15.22	14.91	13.23	12.29	−4.73 (−5.90, −3.54)	−7.66	<0.01

CMR, Crude mortality rate; ASMR, Age-standardized mortality rate; AAPC, Average annual percent change; CI, Confidence interval.

Throughout the study period, the mortality rate for females was consistently lower than that for males. In males, CMR and ASMR decreased from 27.83 and 31.90 per 100,000 in 2008 to 25.10 and 17.91 per 100,000 in 2021, respectively, with a significant decrease in ASMR (AAPC = −4.55, 95% CI: −5.60% to −3.49%; *p* < 0.01). Females not only had a lower mortality rate but also experienced a greater downward trend. CMR and ASMR decreased from 13.13 and 13.44 per 100,000 in 2008 to 11.46 and 7.16 per 100,000 in 2021, respectively (AAPC for ASMR = −5.01, 95% CI: −6.56% to −3.43%; *p* < 0.01).

From 2008 to 2021, the gastric cancer mortality rate was higher in rural areas than in urban areas; however, the decline in ASMR was also greater in rural areas, resulting in a decrease in the urban–rural difference in ASMR year by year. Among rural residents, CMR and ASMR decreased from 21.77 and 25.69 per 100,000 in 2008 to 19.49 and 13.10 per 100,000 in 2021, respectively; AAPC derived from Joinpoint showed a significant decrease in ASMR (−4.92, 95% CI: −5.48% to −4.36%; *p* < 0.01). Among urban residents, CMR and ASMR decreased from 18.55 and 17.56 per 100,000 in 2008 to 16.27 and 10.76 per 100,000 in 2021, respectively (AAPC = −3.99, 95% CI: −5.35% to −2.61%; *p* < 0.01).

Significant regional differences were observed, with gastric cancer mortality rates in Western China lower than in Eastern and Central China. Although mortality rates declined in all three regions, the decrease in ASMR was most significant in Western China, from 21.23 per 100,000 people in 2008 to 10.63 per 100,000 people in 2021, with an AAPC of −5.23% (95% CI: −6.53% to −3.91%; *p* < 0.01). The gastric cancer mortality rate is relatively high in Eastern China and Central China. The ASMR decreased from 21.68/24.52 cases per 100,000 people to 20.76/12.95 cases per 100,000 people in 2021, with AAPCs of −4.10 and −4.94, respectively, and *p*-values of less than 0.01.

### 3.3 Burden and trends of gastric cancer mortality

**Figure 3:**
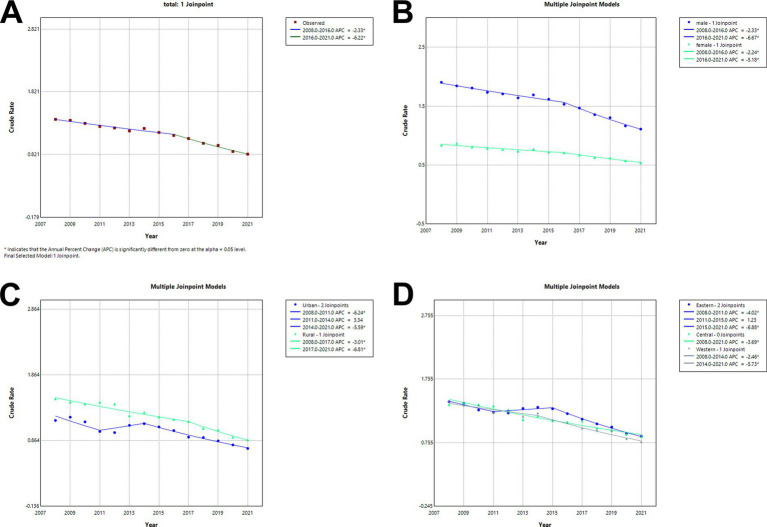
Joinpoint regression of gastric cancer PYLLR trends in China, 2008–2021. Joinpoint regression analyses of PYLLR for gastric cancer from 2008 to 2021. **(A)** Overall population. **(B)** By sex (male and female). **(C)** By residence (urban and rural). **(D)** By region (Eastern, Central, and Western China). APC for each segment is displayed in the legend; an asterisk indicates APC significantly different from zero at *α* = 0.05.

From 2008 to 2021, the PYLLR from gastric cancer in China showed a continuous downward trend. The overall PYLLR decreased from 1.38‰ in 2008 to 1.13‰ in 2016, and further decreased to 0.83‰ in 2021. Joinpoint modeling showed that this trend was statistically significant, with an AAPC of −3.84% (*p* < 0.01) (Table 2; Figure 3; Supplementary Figure 2).

**Table 2:** PYLL and PYLLR of gastric cancer from 2008 to 2021 in China.

Index	Measure	2008	2009	2010	2011	2012	2013	2014	2015	2016	2017	2018	2019	2020	2021	AAPC% (95%CI)	t	*p*
Total	PYLL	102258.50	102527.50	103789.50	98169.00	96138.00	271992.00	314412.50	302850.50	298494.50	291701.50	272065.00	267947.50	243076.00	222014.00			
	PYLLR	1.38	1.37	1.32	1.27	1.25	1.20	1.24	1.18	1.13	1.08	1.00	0.97	0.87	0.83	−3.84(−4.41, −3.27)	−12.93	<0.01
Male	PYLL	71924.00	70621.50	72630.50	68301.50	67194.00	189933.00	218686.50	211443.50	206568.00	202188.00	187671.00	183333.00	164644.50	150999.50			
	PYLLR	1.90	1.84	1.81	1.73	1.71	1.64	1.69	1.62	1.53	1.47	1.36	1.30	1.16	1.11	−4.02(−4.58, −3.46)	−13.75	<0.01
Female	PYLL	30334.50	31906.00	31159.00	29867.50	28944.00	82059.00	95726.00	91407.00	91926.50	89513.50	84394.00	84614.50	78431.50	71014.50			
	PYLLR	0.84	0.87	0.81	0.79	0.76	0.74	0.77	0.72	0.71	0.67	0.63	0.62	0.57	0.54	−3.38(−4.11, −2.64)	−8.89	<0.01
Urban areas	PYLL	30249.50	32491.00	34215.50	30891.00	30283.50	76967.00	90706.50	88802.00	89824.50	83836.00	84502.50	81034.50	77815.50	68100.00			
	PYLLR	1.17	1.22	1.15	1.00	0.98	1.09	1.12	1.07	1.02	0.92	0.91	0.86	0.80	0.74	−3.75(−5.85, −1.60)	−3.40	<0.01
Rural areas	PYLL	72009.00	70036.50	69574.00	67278.00	65854.5	195025.00	223706.00	214048.50	208670.00	207865.50	187562.50	186913.00	165260.50	153914.00			
	PYLLR	1.50	1.45	1.42	1.45	1.42	1.24	1.30	1.23	1.18	1.16	1.05	1.02	0.91	0.87	−4.19(−5.11, −3.26)	−8.70	<0.01
Eastern China	PYLL	39504.50	39504.50	39090.50	37242.50	36759.5	113502.00	130911.00	127431.00	123848.50	120215.50	112637.50	109387.50	98672.00	91577.00			
	PYLLR	1.40	1.38	1.27	1.23	1.223599426	1.29	1.31	1.29	1.21	1.12	1.05	1.00	0.90	0.85	−3.79(−4.80, −2.77)	−7.19	<0.01
Central China	PYLL	34398.00	35732.00	36137.50	36065.50	34,459	92241.00	105433.50	98539.50	100729.50	103815.00	93003.00	90899.50	84552.50	76290.00			
	PYLLR	1.35	1.38	1.36	1.33	1.272319263	1.12	1.18	1.10	1.08	1.09	0.98	0.96	0.89	0.85	−3.69(−4.31, −3.07)	−12.76	<0.01
Western China	PYLL	28356.00	27291.00	28561.50	24861.00	24919.5	66249.00	78068.00	76880.00	73916.50	67671.00	66424.50	67660.50	59851.50	54147.00			
	PYLLR	1.40	1.34	1.34	1.23	1.240375758	1.16	1.21	1.11	1.06	0.97	0.94	0.93	0.81	0.76	−4.23(−5.05, −3.41)	−9.93	<0.01

PYLL, Potential years of life lost; PYLLR (‰), Potential years of life lost rate; AAPC, Average annual percent change; CI, Confidence interval.

Gender-specific analysis revealed that the PYLLR for women was consistently lower than that for men throughout the study period. The female PYLLR decreased significantly from 0.84‰ in 2008 to 0.54‰ in 2021 (AAPC = −3.38%, *p* < 0.01). The male PYLLR decreased more significantly from 1.90‰ in 2008 to 1.11‰ in 2021 (AAPC = −4.02%, *p* < 0.01).

A comparison between urban and rural areas showed that the PYLLR of rural residents was consistently higher than that of urban residents. However, from 2008 to 2021, the PYLLR of both groups decreased significantly over time. The rural PYLLR decreased from 1.50‰ in 2008 to 0.87‰ in 2021 (AAPC = −4.19%, *p* < 0.01). The urban PYLLR was 1.17‰ in 2008, and after a brief rebound from 2011 to 2014, it decreased to 0.74‰ in 2021, but the overall downward trend remained significant (AAPC = −3.75%, *p* < 0.01).

Regional stratified analysis showed that the PYLLR for gastric cancer in China generally showed a continuous downward trend across the eastern, central, and western regions. The PYLLR in the eastern region decreased from 1.40‰ in 2008 to 0.85‰ in 2021; in the central region from 1.35‰ to 0.85‰; and in the western region from 1.40‰ to 0.76‰, with the differences between the three regions narrowing in 2021. Overall AAPC further confirmed statistically significant decreases in all three regions: eastern region (AAPC = −3.79%, *p* < 0.01), central region (AAPC = −3.69%, *p* < 0.01), and western region (AAPC = −4.23%, *p* < 0.01), with the western region showing the largest overall decrease.

### 3.4 The age-period-cohort model analysis of gastric cancer mortality

**Figure 4:**
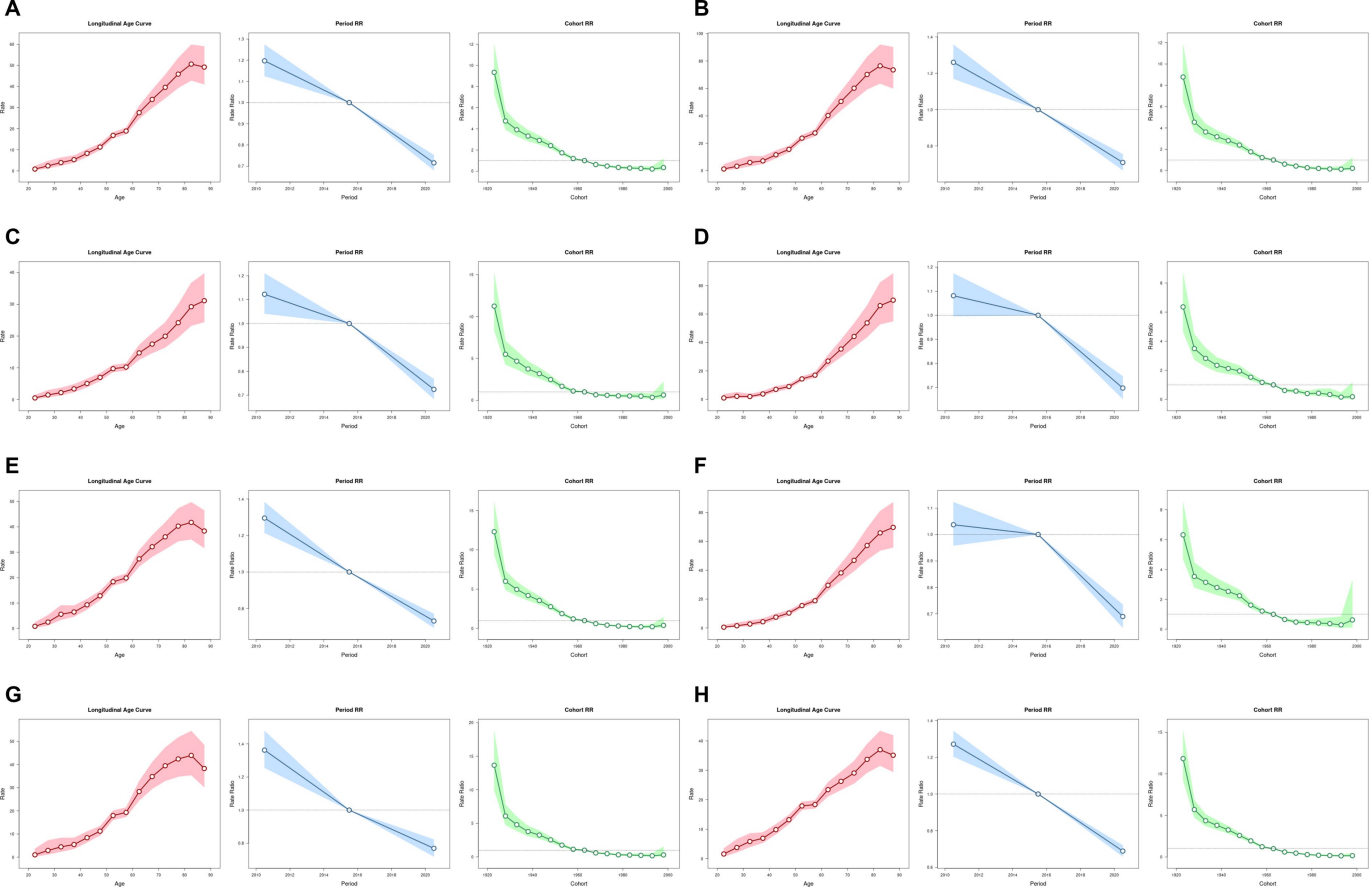
Age–period–cohort effects on gastric cancer mortality in China, 2008–2021. Age–period–cohort analyses were performed using the National Cancer Institute APC Web tool and were restricted to individuals aged 20 years and older. Age groups were represented by five-year age-group midpoints from 22.5 to 87.5 years, calendar periods by midpoints of 2010.5, 2015.5, and 2020.5, and birth cohorts by midpoints from 1923 to 1998. Rate ratios were estimated relative to age 52.5 years, period 2015.5, and birth cohort 1963. Panels are presented for: **(A)** overall population, **(B)** males, **(C)** females, **(D)** rural residents, **(E)** urban residents, **(F)** Eastern China, **(G)** Central China, and **(H)** Western China.

Overall, the age effect, period effect, and cohort effect remained largely consistent across the general population and subgroups (Figure 4; Supplementary Table S1). Since the gastric cancer mortality rate is near zero before age 20, this study only included the ≥20-year age group in the age-period-cohort effect analysis for better visualization. The age effect showed a gradually increasing trend in the longitudinal age curve, accelerating after approximately age 60. After adjustment for age and birth cohort, the period RRs declined over time, with the total population RR decreasing from 1.20 in 2010.5 to 0.72 in 2020.5 relative to the reference period of 2015.5. Cohort RRs also showed a marked downward pattern across successive birth cohorts, decreasing from 9.35 in the 1923 birth cohort to 0.34 in the 1998 birth cohort relative to the 1963 reference cohort. The net drift for total gastric cancer mortality was −5.02% per year (95% CI: −5.87% to −4.15%), and negative net drifts were observed in all sex, residence, and regional subgroups, suggesting an overall downward mortality trend after accounting for age, period, and cohort dimensions. Key APC model estimates are summarized in Supplementary Table S1.

### 3.5 Gastric cancer mortality by age group

**Figure 5:**
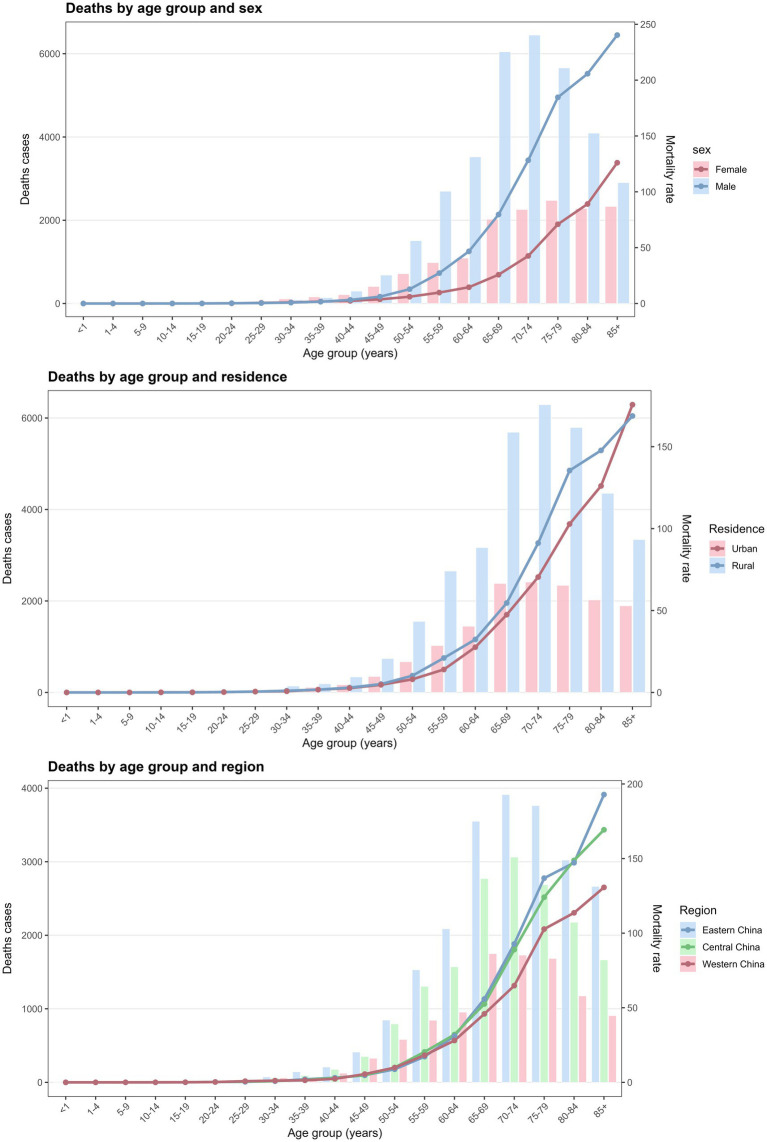
Age-specific deaths and CMR of gastric cancer in 2021, stratified by sex, residence, and region. Age-specific gastric cancer deaths and CMR in 2021. **(A)** Stratified by sex (male vs. female). **(B)** Stratified by residence (rural vs. urban). **(C)** Stratified by region (Eastern, Central, and Western China). Mortality rates are presented per 100,000 population.

Figure 5 shows age-specific deaths and age-specific CMR stratified by sex, residence, and region. In 2021, the CMR of gastric cancer in all subgroups showed an increasing trend with age. The number of gastric cancer deaths in each subgroup initially increased with age, peaking between 65 and 79 years, and then declined. In all age groups, the number of gastric cancer deaths and the CMR were higher in men than in women. The number of gastric cancer deaths and the CMR in Eastern China and Central China were higher than in Western China. In the age group under 85 years, the number of gastric cancer deaths and the CMR were higher in rural areas than in urban areas; only in the age group over 85 years was the CMR of gastric cancer higher in urban areas than in rural areas.

**Figure 6:**
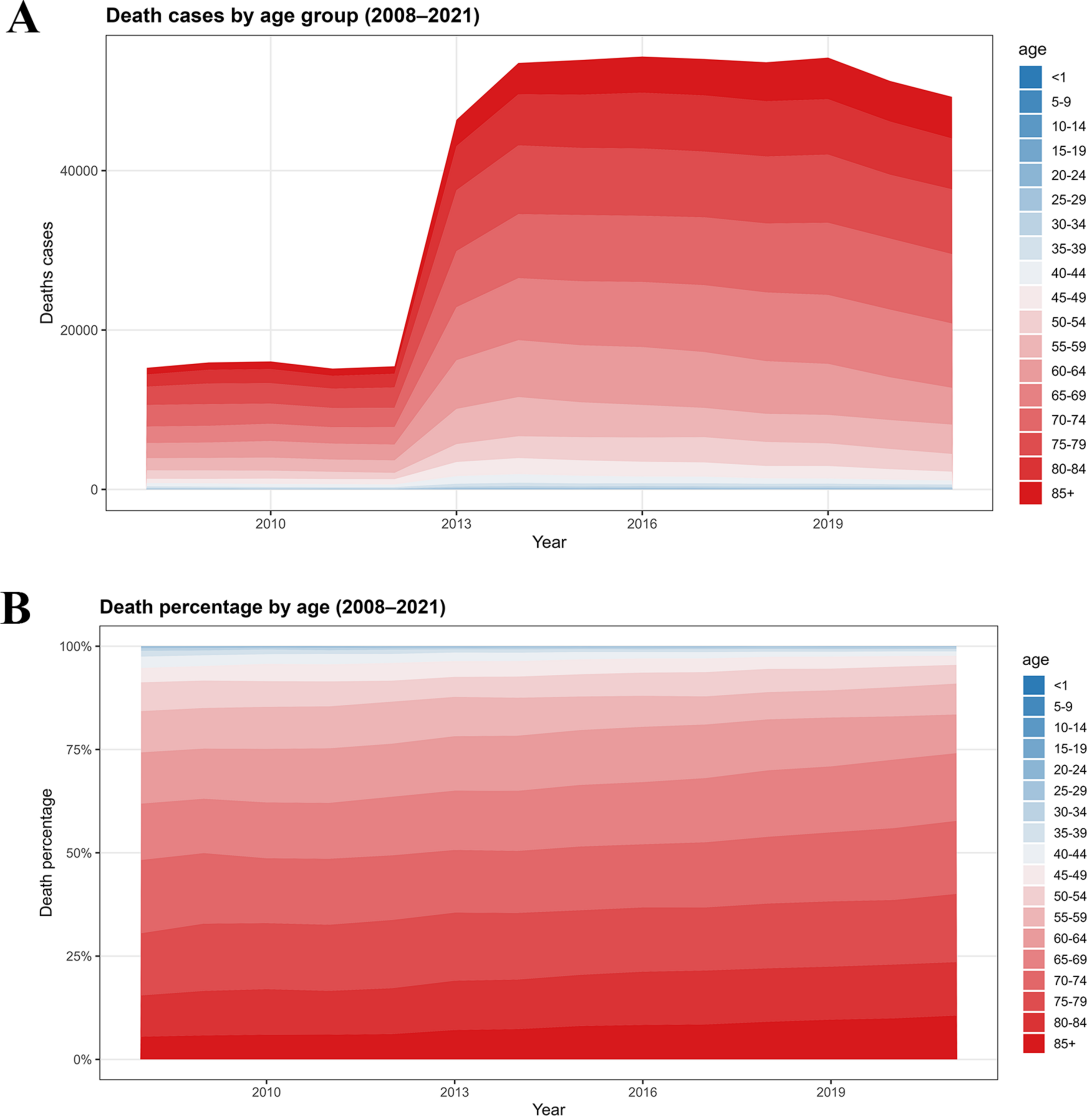
Temporal changes in gastric cancer deaths by age group in China, 2008–2021. **(A)** Stacked area plot of the number of gastric cancer deaths by age group from 2008 to 2021. **(B)** Stacked proportional area plot showing the age composition (percentage) of gastric cancer deaths over the same period.

Between 2008 and 2021, the number of age-specific deaths exhibited significant temporal variations and age gradient characteristics (Figure 6A). From 2008 to 2012, the overall number of deaths remained at a low level with little fluctuation. From 2013 onwards, the number of deaths showed a significant step increase, remained at a high level in subsequent years, and then slightly declined in recent years. The surge in 2012–2013 was closely related to the expansion of China’s cause-of-death surveillance system in 2013. The increased number of surveillance sites improved the capture and reporting of mortality events, resulting in a structural break in the absolute mortality figures over time. Regarding the contribution of each age group, the number of deaths increased rapidly with age, with the mortality burden mainly concentrated in the middle-aged and older populations, especially the ≥60-year-old age group, while the number of deaths in younger populations (<40 years old) remained consistently low.

Figure 6B further characterizes the age structure changes using the mortality composition ratio. In each year, deaths were mainly composed of older individuals, with the proportion of younger individuals remaining consistently low, indicating a high concentration of mortality burden in the older adults. Unlike the absolute mortality figures, the mortality composition ratio was more stable around 2013, suggesting that the expansion of the surveillance system had a relatively limited impact on the age structure. From a temporal trend perspective, from 2008 to 2021, the overall composition ratio of gastric cancer deaths in China showed a structural characteristic of concentration towards the older age group (≥60 years old).

### 3.6 Projection of gastric cancer mortality to 2036

**Figure 7:**
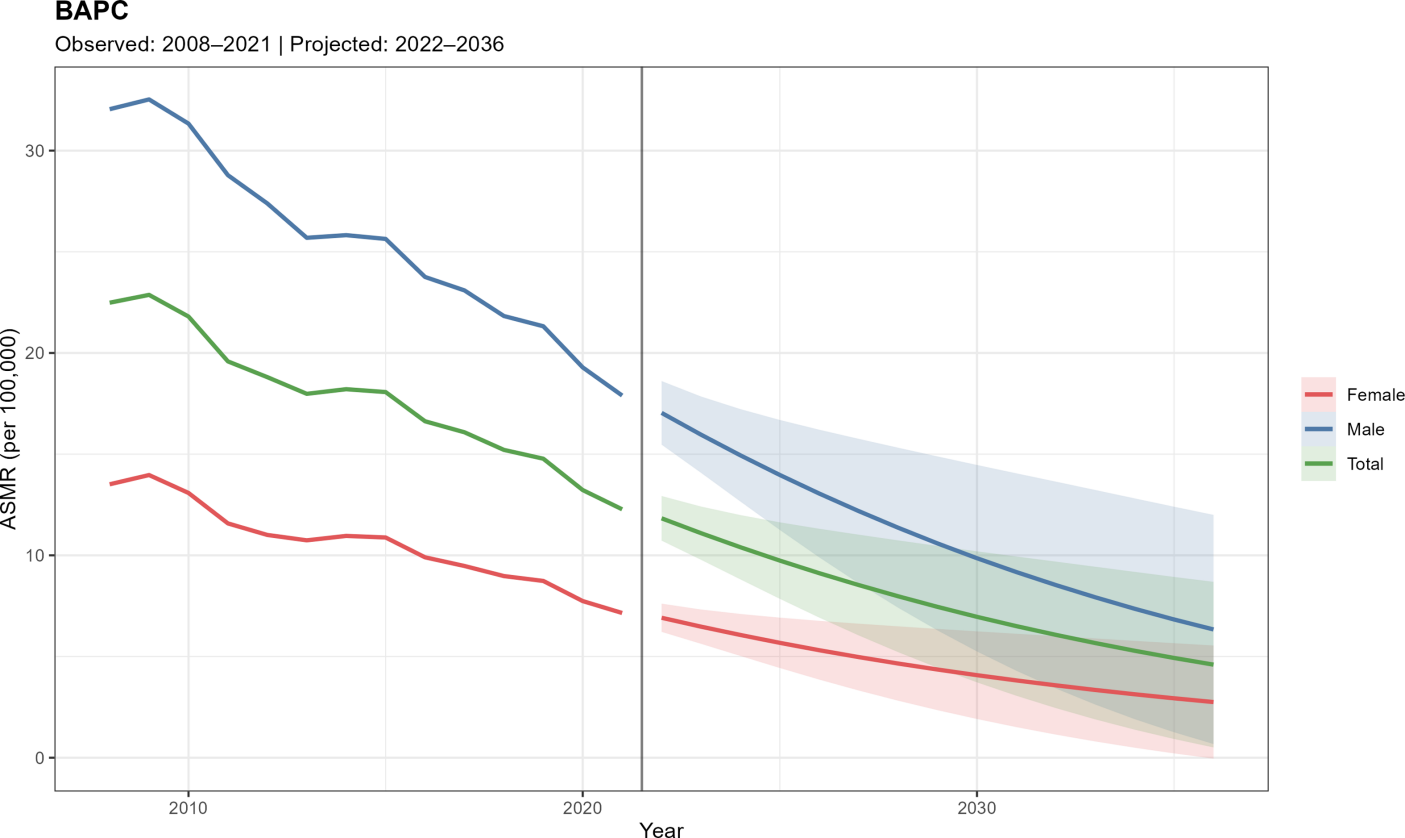
BAPC projection of gastric cancer ASMR by sex in China, 2022–2036. BAPC projections of ASMR for gastric cancer in China. Solid lines show observed ASMRs for 2008–2021 and projected ASMRs for 2022–2036 for the total population and by sex (male and female). Shaded bands represent 95% credible intervals. The vertical line separates the observed period from the projection period. ASMRs were standardized to the 2010 Chinese standard population. BAPC models used second-order random-walk smoothing priors for age, period, and cohort effects and projected population denominators from the Institute for Health Metrics and Evaluation.

Predictions of future burden based on the BAPC model show a continuous decline in ASMR in both the general population and both sexes between 2022 and 2036. In the total population, the projected ASMR decreased from 12.27 per 100,000 in 2021 to 6.96 per 100,000 in 2030 and 4.60 per 100,000 in 2036. The corresponding projected ASMRs were higher in males than females throughout the projection period: 9.86 versus 4.08 per 100,000 in 2030, and 6.34 versus 2.75 per 100,000 in 2036. Uncertainty widened over longer projection horizons, particularly in later years (Figure 7; Supplementary Table S2). In back-testing, the model showed acceptable short-term predictive performance. For the total population, the MAE was 1.30 per 100,000 and the MAPE was 9.84%; all four observed ASMRs for 2018–2021 fell within the 95% credible intervals. Similar performance was observed in males (MAE 1.21 per 100,000, MAPE 6.42%, coverage 4/4) and females (MAE 0.69 per 100,000, MAPE 9.03%, coverage 4/4) (Supplementary Table S3).

### 3.7 Sensitivity analysis

In sensitivity analysis restricted to 2013–2021, the principal findings remained consistent with the main analyses. During the post-expansion period, ASMR continued to decline overall, with an APC of −4.85. Declining trends were also observed for PYLLR, with an APC of −4.90. The major subgroup patterns were preserved: mortality remained higher in males than in females, higher in rural than in urban populations, and regional variation persisted across eastern, central, and western China (Supplementary Figures 3, 4). Overall, these restricted-period analyses supported the direction and interpretation of the main results.

In the sensitivity analysis based on rate-based post-stratified national estimates, both estimated deaths and ASMRs showed overall declining trends from 2008 to 2021. The estimated total number of gastric cancer deaths decreased from 315,189 in 2008 to 250,465 in 2021. The total ASMR decreased from 22.57 to 12.31 per 100,000 population over the same period. Sex-specific patterns were consistent with the main analysis. Males consistently had higher estimated deaths and ASMRs than females throughout the study period. Male estimated deaths decreased from 219,421 in 2008 to 174,146 in 2021, while male ASMR declined from 31.91 to 17.90 per 100,000 population. Female estimated deaths decreased from 95,768 to 76,319, and female ASMR declined from 13.45 to 7.16 per 100,000 population. Importantly, after applying the post-stratification approach, no marked step increase was observed around 2013. This suggests that the declining patterns in gastric cancer mortality were not primarily driven by the expansion of DSP surveillance coverage in 2013 (Supplementary Table S4; Supplementary Figure 5).

The sensitivity analysis using 75 years as the PYLL threshold yielded findings consistent with the main analysis based on the 70-year threshold. In the total population, PYLLR decreased from 1.83 per 1,000 population in 2008 to 1.17 per 1,000 population in 2021, with a significant AAPC of −3.36% (95% CI: −3.69% to −2.95%; *p* < 0.01). Declining trends were also observed in males (AAPC = −3.44, 95% CI: −3.98% to −2.90%), females (AAPC = −3.10, 95% CI: −3.80% to −2.39%), urban areas (AAPC = −3.32, 95% CI: −5.63% to −0.94%), rural areas (AAPC = −3.70, 95% CI: −4.57% to −2.83%), eastern China (AAPC = −3.27, 95% CI: −4.30% to −2.23%), central China (AAPC = −3.29, 95% CI: −3.85% to −2.72%), and western China (AAPC = −3.82, 95% CI: −4.65% to −2.99%) (Supplementary Table S5; Supplementary Figure 6). These results support the robustness of the observed decline in premature mortality burden.

## 4 Discussion

This nationwide study based on China’s Disease Surveillance Points system provides an updated surveillance-based assessment of gastric cancer mortality, premature mortality burden, demographic disparities, and future trends in mainland China from 2008 to 2021. Several major findings emerged. First, gastric cancer mortality declined substantially over the study period, with a much larger reduction in ASMR than in CMR. Second, important disparities persisted, with consistently higher mortality and premature mortality burden in males and rural residents. Third, regional variation remained evident, although all three major regions experienced declining mortality. Fourth, both the age-period-cohort analysis and the age-specific mortality structure indicated that the burden of gastric cancer was increasingly concentrated among older adults. Sensitivity analyses and model diagnostics generally supported the robustness of the main rate-based findings, although absolute death counts should be interpreted cautiously because of changes in DSP system coverage.

Between 2008 and 2021, China’s age-adjusted mortality risk steadily declined, with the rate of decrease accelerating after 2019. However, the reduction in the CMR was more modest, indicating that population aging partially offset these gains due to the growing number of older adults at high risk of death from gastric cancer. In 2019, China issued the Healthy China Action Cancer Prevention and Control Implementation Plan (2019–2022), which emphasized cancer screening, early diagnosis and treatment, expansion of screening coverage, and establishment of long-term screening mechanisms. The post-2019 decline observed in our analysis is chronologically consistent with this policy.

This study observed significant spatial heterogeneity in the risk of death from gastric cancer: the ASMR was generally higher in the eastern and central regions than in the western region, and remained relatively higher at both the beginning and end of the study period. The observed heterogeneity may reflect a combination of demographic structure, competing mortality patterns, regional differences in risk-factor distributions, diagnostic practice, treatment access, and reporting quality (16, 17). Previous national risk-factor surveillance has shown substantial geographic variation in obesity in China; for example, adult general obesity in 2013–2014 was estimated at 14.0% nationally, and provincial prevalence varied widely, reaching more than 20% in some northern municipalities (18, 19). Obesity has been more consistently linked to cardia gastric cancer than to non-cardia gastric cancer, but the DSP mortality data used in the present study were based on ICD-10 code C16 for overall gastric cancer and did not provide sufficiently reliable subtype-specific information to distinguish cardia from non-cardia gastric cancer mortality. Therefore, obesity-related subtype differences can only be considered as a possible contextual factor and cannot be directly evaluated in this study. Future, more refined spatial-scale analyses (at the provincial, municipal, and county levels) will help identify high-risk clusters, thereby improving the accuracy of resource allocation.

In this study, CMR, ASMR, and PYLLR for gastric cancer were consistently higher in males than in females. Previous studies have reported substantially higher gastric cancer incidence in males than females, which is consistent with the higher mortality and premature mortality burden observed among males in this study (20). Biological mechanisms, including potential protective effects of estrogen, have been proposed as one possible explanation for sex differences in gastric cancer risk (21). Estrogen can reduce carcinogenic damage to the gastric mucosa by promoting DNA repair, improving mucosal defense, and reducing chronic inflammation (22). Finally, males are more exposed to smoking, high-salt diet, and alcohol consumption, which are associated with higher gastric cancer incidence and mortality (23, 24). The persistence of gender differences suggests that primary prevention (such as diet, smoking, and drinking-related interventions, risk factor control), high-risk screening, and early diagnosis and treatment for middle-aged and older males should still be the priority direction of prevention and control strategies.

In terms of urban–rural differences, the mortality rate and burden in rural areas consistently exceeded those in urban areas during the study period. According to previous studies, rural residence itself is an independent factor associated with high mortality from gastric cancer (25). This pattern is consistent with previous evidence that rural populations may experience higher exposure to established gastric cancer risk factors, including *Helicobacter pylori* infection, high-salt preserved foods, smoking, and alcohol use (26, 27), as well as potential delays in diagnosis and differences in access to high-quality medical services (28, 29). On the other hand, the study found that the gap between urban and rural areas is smaller in 2021 than in 2008. We hypothesize that this means that the improvement of public health and medical service capabilities in recent years has produced greater marginal effects in rural areas, including the improvement of medical insurance coverage and assistance systems, and the enhancement of primary medical service capabilities.

This study further explained the changes in gastric cancer death risk from three dimensions: age, period, and birth cohort. The age effect shows that the risk of death from gastric cancer increases with age and accelerates after about the age of 60, consistent with the accumulation of carcinogens (30), the accumulation of cancer-causing mutations caused by aging itself (31), and poor treatment tolerance (32). The period effect continued to decline after controlling for age and cohort, which may be related to China’s continued efforts in the prevention and control of gastric cancer in recent years. In 2009, China took the lead in successfully developing an oral recombinant Hp vaccine (33). China launched a rural cancer screening project in 2005 and an urban cancer screening project in 2012 to strengthen early diagnosis and treatment of gastric cancer; approximately two million high-risk individuals had been screened by the end of 2016 (34). The cohort effect shows that the risk is higher in the early birth cohort, and then continues to decline but gradually approaches a plateau as the birth years go by. This may reflect that improvements in long-term living environment and early life course exposure have contributed to the risk reduction, but further decline may face a marginal decline, requiring more targeted primary prevention and high-risk screening strategies.

The restricted-period sensitivity analysis for 2013–2021 provides additional support for the robustness of the main findings. Because this period represents the post-expansion phase of the DSP system, with broader coverage and greater surveillance stability, the consistency of declining ASMR and PYLLR and the persistence of subgroup disparities within this interval suggest that the overall conclusions were not solely driven by the 2013 system expansion. At the same time, the pre-expansion period (2008–2012) was not emphasized as the primary basis for sensitivity inference because it contained only five annual observations, which limited the stability and precision of subgroup-specific trend estimates. A supplementary sensitivity analysis using rate-based post-stratified national estimates further supported the robustness of our findings. After applying DSP-derived age- and sex-specific mortality rates to national population denominators, estimated deaths and ASMRs continued to decline, and no marked step increase was observed around 2013. Taken together, these findings support the interpretation that the observed decline in age-adjusted mortality risk and premature mortality burden, as well as the persistence of demographic and regional disparities, remained broadly robust in the post-expansion surveillance era.

This study provides a comprehensive national assessment and prediction of gastric cancer-related mortality and premature death burden in mainland China, offering demographic evidence for the cumulative impact of gastric cancer prevention and treatment. However, this study has several limitations. First, although the DSP system provides an important national mortality surveillance resource, the expansion of surveillance points in 2013 may affect the comparability of absolute death counts over time. We therefore emphasized rate-based indicators when interpreting temporal trends. Second, the APC analysis was based on a log-linear Poisson modeling framework. Although this approach is widely used for cancer incidence and mortality surveillance, potential overdispersion, ecological bias, and model misspecification cannot be fully excluded. In addition, because age, period, and cohort are linearly dependent, APC estimates should be interpreted cautiously and should not be used to infer individual-level causality. Third, the DSP mortality data were based on overall gastric cancer deaths coded as ICD-10 C16 and did not provide sufficiently detailed information to distinguish cardia and non-cardia gastric cancer mortality. Because these subtypes differ in etiology and epidemiologic patterns, combining them may mask subtype-specific trends and may limit interpretation of obesity-related or other risk-factor explanations. Fourth, regional, urban–rural, and sex differences were analyzed using aggregated surveillance data; therefore, ecological explanations involving risk factors, screening, medical resources, or treatment access should be interpreted as hypotheses rather than causal conclusions. Fifth, the BAPC projections assume continuation of recent age-period-cohort patterns and do not explicitly account for future changes in risk factors, screening, diagnosis, treatment, or coding practice; uncertainty therefore increases over longer.

## 5 Conclusion

Using nationally representative DSP surveillance data from 2008 to 2021, this study provides an integrated assessment of gastric cancer mortality, premature mortality burden, demographic disparities, and future trends in mainland China. We observed substantial declines in both ASMR and PYLLR over time; however, marked disparities persisted, with higher mortality and premature mortality burden in males and rural residents and continued regional variation across China. More importantly, the much larger decline in ASMR than in CMR, together with the increasing concentration of gastric cancer deaths among older adults, suggests that population ageing continues to reshape the burden of gastric cancer despite improving age-adjusted mortality risk. These findings support sustained and more equitable gastric cancer control strategies, with particular emphasis on high-risk groups such as older men and rural populations.

## Data Availability

The original contributions presented in the study are included in the article/Supplementary material, further inquiries can be directed to the corresponding author.

## Footnotes

1
https://analysistools.cancer.gov/apc/
