# Supplementary material for: Population ageing and gastric cancer mortality burden in China, 2008–2021: a nationwide analysis with projections to 2036
Source: Front Public Health. 2026 Aug 5;14:1875254. doi: 10.3389/fpubh.2026.1875254 (PMC13485546; doi:10.3389/fpubh.2026.1875254)
Supplement: Supplementary file 1 [file Supplementary_file_1.DOCX]

**Supplementary Figure 1. Joinpoint regression of gastric cancer ASMR trends**

**Supplementary Figure 2. Joinpoint regression of gastric cancer PYLLR trends**

**Supplementary Figure 3.**

**Joinpoint regression of gastric cancer ASMR trends in China, 2013–2021.**

**Supplementary Figure 4.**

**Joinpoint regression of gastric cancer PYLLR trends in China, 2013–2021.**

**Supplementary Figure 5.**

**Historical and projected deaths and ASMRs of cerebrovascular disease 2008-2021.**

Bars indicate annual numbers of deaths (left y-axis), and lines indicate ASMRs (per 100,000 population, right y-axis) for the total population, females, and males.

**Supplementary Figure 6.**

**Joinpoint regression of gastric cancer PYLLR（75-year threshold） trends in China, 2008–2021.**

**Supplementary Table S1.**

**Age-period-cohort model estimates of gastric cancer mortality in China, 2008-2021**

| Group | Net drift, %/year (95% CI) | Period RR 2010.5 (95% CI) | Period RR 2015.5 | Period RR 2020.5 (95% CI) | Cohort RR 1923 (95% CI) | Cohort RR 1963 | Cohort RR 1998 (95% CI) | Wald test for period RR, p | Wald test for cohort RR, p |
| --- | --- | --- | --- | --- | --- | --- | --- | --- | --- |
| Total | -5.02 (-5.87, -4.15) | 1.20 (1.13, 1.27) | 1.00 (reference) | 0.72 (0.68, 0.75) | 9.35 (7.31, 11.96) | 1.00 (reference) | 0.34 (0.10, 1.18) | <0.001 | <0.001 |
| Male | -5.58 (-6.66, -4.50) | 1.26 (1.17, 1.36) | 1.00 (reference) | 0.71 (0.67, 0.75) | 8.78 (6.53, 11.81) | 1.00 (reference) | 0.22 (0.04, 1.18) | <0.001 | <0.001 |
| Female | -4.29 (-5.25, -3.31) | 1.12 (1.04, 1.21) | 1.00 (reference) | 0.72 (0.68, 0.77) | 11.24 (8.34, 15.14) | 1.00 (reference) | 0.63 (0.18, 2.19) | <0.001 | <0.001 |
| Urban areas | -4.28 (-5.43, -3.12) | 1.08 (1.00, 1.17) | 1.00 (reference) | 0.70 (0.65, 0.75) | 6.36 (4.67, 8.66) | 1.00 (reference) | 0.18 (0.03, 1.16) | <0.001 | <0.001 |
| Rural areas | -5.56 (-6.45, -4.66) | 1.30 (1.21, 1.38) | 1.00 (reference) | 0.73 (0.69, 0.77) | 12.30 (9.43, 16.06) | 1.00 (reference) | 0.40 (0.11, 1.44) | <0.001 | <0.001 |
| Eastern China | -3.99 (-5.12, -2.85) | 1.04 (0.96, 1.12) | 1.00 (reference) | 0.69 (0.65, 0.74) | 6.32 (4.70, 8.50) | 1.00 (reference) | 0.62 (0.12, 3.26) | <0.001 | <0.001 |
| Central China | -5.55 (-6.68, -4.41) | 1.36 (1.26, 1.48) | 1.00 (reference) | 0.77 (0.72, 0.82) | 13.66 (9.94, 18.76) | 1.00 (reference) | 0.30 (0.06, 1.52) | <0.001 | <0.001 |
| Western China | -5.94 (-6.66, -5.22) | 1.27 (1.20, 1.34) | 1.00 (reference) | 0.69 (0.66, 0.72) | 11.83 (9.24, 15.15) | 1.00 (reference) | 0.16 (0.05, 0.46) | <0.001 | <0.001 |

Notes: All APC models were fitted using the National Cancer Institute age-period-cohort tool within a Poisson regression framework. Rate ratios were estimated relative to age 52.5 years, period 2015.5, and birth cohort 1963. Age groups were represented by five-year midpoints from 22.5 to 87.5 years, periods by 2010.5, 2015.5, and 2020.5, and birth cohorts by 1923-1998. Net drift represents the overall annual percentage change in the fitted age-adjusted rates. RR, rate ratio; CI, confidence interval.

**Supplementary Table S2**

**Sex-specific projected ASMRs with 95% CI using the BAPC model, 2022-2036**

| Group | Year | Lower | Mean | Upper |
| --- | --- | --- | --- | --- |
| Female | 2022 | 6.22 | 6.92 | 7.61 |
| Female | 2023 | 5.63 | 6.48 | 7.32 |
| Female | 2024 | 5.03 | 6.06 | 7.10 |
| Female | 2025 | 4.43 | 5.68 | 6.92 |
| Female | 2026 | 3.86 | 5.31 | 6.76 |
| Female | 2027 | 3.32 | 4.97 | 6.62 |
| Female | 2028 | 2.82 | 4.65 | 6.49 |
| Female | 2029 | 2.35 | 4.36 | 6.36 |
| Female | 2030 | 1.91 | 4.08 | 6.24 |
| Female | 2031 | 1.51 | 3.82 | 6.12 |
| Female | 2032 | 1.14 | 3.58 | 6.01 |
| Female | 2033 | 0.80 | 3.35 | 5.89 |
| Female | 2034 | 0.49 | 3.13 | 5.77 |
| Female | 2035 | 0.21 | 2.93 | 5.66 |
| Female | 2036 | -0.04 | 2.75 | 5.54 |
| Male | 2022 | 15.47 | 17.04 | 18.61 |
| Male | 2023 | 14.08 | 15.96 | 17.84 |
| Male | 2024 | 12.66 | 14.94 | 17.22 |
| Male | 2025 | 11.26 | 13.97 | 16.68 |
| Male | 2026 | 9.90 | 13.05 | 16.19 |
| Male | 2027 | 8.62 | 12.18 | 15.74 |
| Male | 2028 | 7.42 | 11.36 | 15.30 |
| Male | 2029 | 6.29 | 10.58 | 14.87 |
| Male | 2030 | 5.26 | 9.85 | 14.45 |
| Male | 2031 | 4.30 | 9.17 | 14.03 |
| Male | 2032 | 3.43 | 8.53 | 13.62 |
| Male | 2033 | 2.63 | 7.92 | 13.21 |
| Male | 2034 | 1.91 | 7.35 | 12.79 |
| Male | 2035 | 1.27 | 6.82 | 12.38 |
| Male | 2036 | 0.68 | 6.33 | 11.98 |
| Total | 2022 | 10.73 | 11.83 | 12.93 |
| Total | 2023 | 9.78 | 11.10 | 12.41 |
| Total | 2024 | 8.81 | 10.40 | 11.99 |
| Total | 2025 | 7.85 | 9.74 | 11.63 |
| Total | 2026 | 6.92 | 9.12 | 11.31 |
| Total | 2027 | 6.04 | 8.53 | 11.02 |
| Total | 2028 | 5.22 | 7.98 | 10.74 |
| Total | 2029 | 4.44 | 7.45 | 10.46 |
| Total | 2030 | 3.72 | 6.96 | 10.20 |
| Total | 2031 | 3.06 | 6.50 | 9.94 |
| Total | 2032 | 2.45 | 6.07 | 9.69 |
| Total | 2033 | 1.89 | 5.67 | 9.44 |
| Total | 2034 | 1.39 | 5.28 | 9.18 |
| Total | 2035 | 0.92 | 4.93 | 8.93 |
| Total | 2036 | 0.51 | 4.60 | 8.69 |

ASMR, Age-standardized mortality rate; CI, Confidence interval; BAPC, Bayesian age-period-cohort.

**Supplementary Table S3.**

**BAPC backtesting results from 2018 to 2021**

| Group | Year | observed_asmr | lower | mean | upper | abs_error | ape_percent | MAE | MAPE_percent | covered_95ci |
| --- | --- | --- | --- | --- | --- | --- | --- | --- | --- | --- |
| Total | 2018 | 15.21 | 14.71 | 15.86 | 17.00 | 0.65 | 4.27 | 0.69 | 9.03 | TRUE |
| Total | 2019 | 14.79 | 13.90 | 15.54 | 17.18 | 0.75 | 5.10 |  |  | TRUE |
| Total | 2020 | 13.22 | 12.84 | 15.01 | 17.18 | 1.78 | 13.49 |  |  | TRUE |
| Total | 2021 | 12.28 | 11.62 | 14.30 | 16.99 | 2.02 | 16.49 |  |  | TRUE |
| Male | 2018 | 21.83 | 20.37 | 22.38 | 24.39 | 0.55 | 2.50 | 1.21 | 6.42 | TRUE |
| Male | 2019 | 21.35 | 19.24 | 21.65 | 24.07 | 0.30 | 1.41 |  |  | TRUE |
| Male | 2020 | 19.27 | 18.01 | 20.94 | 23.88 | 1.67 | 8.68 |  |  | TRUE |
| Male | 2021 | 17.90 | 16.71 | 20.24 | 23.76 | 2.34 | 13.06 |  |  | TRUE |
| Female | 2018 | 8.97 | 8.44 | 9.30 | 10.17 | 0.33 | 3.69 | 1.30 | 9.84 | TRUE |
| Female | 2019 | 8.75 | 7.90 | 9.01 | 10.13 | 0.26 | 2.99 |  |  | TRUE |
| Female | 2020 | 7.74 | 7.28 | 8.70 | 10.13 | 0.96 | 12.38 |  |  | TRUE |
| Female | 2021 | 7.15 | 6.61 | 8.37 | 10.14 | 1.22 | 17.05 |  |  | TRUE |

**Supplementary Table S4**

**Deaths and ASMR of gastric cancer from 2008 to 2021**

| year | sex | segment | deaths | cmr | asmr |
| --- | --- | --- | --- | --- | --- |
| 2008 | Total | Observed | 315189 | 23.92 | 22.57 |
| 2009 | Total | Observed | 330492 | 24.94 | 23.22 |
| 2010 | Total | Observed | 294006 | 22.06 | 22.06 |
| 2011 | Total | Observed | 272162 | 20.3 | 19.85 |
| 2012 | Total | Observed | 267535 | 19.84 | 18.86 |
| 2013 | Total | Observed | 264372 | 19.5 | 17.95 |
| 2014 | Total | Observed | 277763 | 20.37 | 18.2 |
| 2015 | Total | Observed | 285756 | 20.84 | 18.04 |
| 2016 | Total | Observed | 273346 | 19.82 | 16.62 |
| 2017 | Total | Observed | 275291 | 19.85 | 16.07 |
| 2018 | Total | Observed | 270840 | 19.42 | 15.15 |
| 2019 | Total | Observed | 270352 | 19.28 | 14.77 |
| 2020 | Total | Observed | 259273 | 18.39 | 13.27 |
| 2021 | Total | Observed | 250465 | 17.67 | 12.31 |
| 2008 | Male | Observed | 219421 | 32.53 | 31.91 |
| 2009 | Male | Observed | 227397 | 33.52 | 32.73 |
| 2010 | Male | Observed | 201449 | 29.52 | 31.62 |
| 2011 | Male | Observed | 188919 | 27.53 | 28.95 |
| 2012 | Male | Observed | 187236 | 27.13 | 27.31 |
| 2013 | Male | Observed | 182222 | 26.25 | 25.65 |
| 2014 | Male | Observed | 190487 | 27.29 | 25.84 |
| 2015 | Male | Observed | 195485 | 27.85 | 25.67 |
| 2016 | Male | Observed | 188239 | 26.67 | 23.76 |
| 2017 | Male | Observed | 190495 | 26.84 | 23.12 |
| 2018 | Male | Observed | 186948 | 26.2 | 21.84 |
| 2019 | Male | Observed | 185902 | 25.91 | 21.36 |
| 2020 | Male | Observed | 179827 | 24.93 | 19.27 |
| 2021 | Male | Observed | 174146 | 24.01 | 17.9 |
| 2008 | Female | Observed | 95768 | 14.9 | 13.45 |
| 2009 | Female | Observed | 103095 | 15.94 | 14.13 |
| 2010 | Female | Observed | 92557 | 14.23 | 13.19 |
| 2011 | Female | Observed | 83243 | 12.72 | 11.55 |
| 2012 | Female | Observed | 80299 | 12.2 | 10.93 |
| 2013 | Female | Observed | 82150 | 12.41 | 10.72 |
| 2014 | Female | Observed | 87276 | 13.11 | 10.99 |
| 2015 | Female | Observed | 90271 | 13.48 | 10.9 |
| 2016 | Female | Observed | 85107 | 12.64 | 9.92 |
| 2017 | Female | Observed | 84796 | 12.53 | 9.48 |
| 2018 | Female | Observed | 83892 | 12.32 | 8.98 |
| 2019 | Female | Observed | 84450 | 12.34 | 8.76 |
| 2020 | Female | Observed | 79446 | 11.54 | 7.75 |
| 2021 | Female | Observed | 76319 | 11.03 | 7.16 |

**Supplementary Table S5**

**Sensitivity analyses of PYLL and PYLLR of gastric cancer from 2008 to 2021.**

| Supplementary Table S5. Sensitivity analyses of PYLL and PYLLR of gastric cancer from 2008 to 2021 | P |  | **<0.01** |  | **<0.01** |  | **<0.01** |  | **<0.01** |  | **<0.01** |  | **<0.01** |  | **<0.01** |  | **<0.01** |
| --- | --- | --- | --- | --- | --- | --- | --- | --- | --- | --- | --- | --- | --- | --- | --- | --- | --- |
|  | t |  | -11.73 |  | -12.21 |  | -8.51 |  | -2.73 |  | -8.18 |  | -6.08 |  | -12.49 |  | -8.87 |
|  | AAPC% (95%CI) |  | -3.36(-3.69, -2.95) |  | -3.44(-3.98, -2.90) |  | -3.10(-3.80, -2.39) |  | -3.32(-5.63, -0.94) |  | -3.70(-4.57, -2.83) |  | -3.27(-4.30, -2.23) |  | -3.29(-3.85, -2.72) |  | -3.82(-4.65, -2.99) |
|  | 2021 | 312275.00 | 1.17 | 216560.00 | 1.59 | 95715.00 | 0.73 | 94840.00 | 1.03 | 217435.00 | 1.23 | 130525.00 | 1.22 | 107511.00 | 1.20 | 74239.00 | 1.05 |
|  | 2020 | 339852.00 | 1.22 | 234749.00 | 1.66 | 105103.00 | 0.77 | 108448.00 | 1.11 | 231404.00 | 1.28 | 139677.00 | 1.28 | 118610.00 | 1.24 | 81565.00 | 1.10 |
|  | 2019 | 371442.00 | 1.34 | 258261.00 | 1.84 | 113181.00 | 0.83 | 111970.00 | 1.18 | 259472.00 | 1.42 | 153164.00 | 1.40 | 126490.00 | 1.33 | 91788.00 | 1.26 |
|  | 2018 | 375872.00 | 1.38 | 262679.00 | 1.90 | 113193.00 | 0.85 | 116397.00 | 1.25 | 259475.00 | 1.45 | 157378.00 | 1.47 | 128498.00 | 1.36 | 89996.00 | 1.28 |
|  | 2017 | 398412.00 | 1.47 | 279595.00 | 2.03 | 118817.00 | 0.89 | 113899.00 | 1.24 | 284513.00 | 1.58 | 166223.00 | 1.56 | 141883.00 | 1.50 | 90306.00 | 1.30 |
|  | 2016 | 406197.00 | 1.53 | 284318.00 | 2.11 | 121879.00 | 0.94 | 121955.00 | 1.38 | 284242.00 | 1.61 | 170427.00 | 1.67 | 137031.00 | 1.47 | 98739.00 | 1.42 |
|  | 2015 | 410874.00 | 1.60 | 289343.00 | 2.21 | 121531.00 | 0.96 | 119912.00 | 1.45 | 290962.00 | 1.67 | 174261.00 | 1.76 | 134075.00 | 1.50 | 102538.00 | 1.48 |
|  | 2014 | 423240.00 | 1.67 | 297355.00 | 2.30 | 125885.00 | 1.01 | 121242.00 | 1.50 | 301998.00 | 1.75 | 177486.00 | 1.78 | 142297.00 | 1.59 | 103457.00 | 1.61 |
|  | 2013 | 366083.00 | 1.61 | 258101.00 | 2.23 | 107982.00 | 0.97 | 102726.00 | 1.46 | 263357.00 | 1.68 | 153442.00 | 1.75 | 124817.00 | 1.51 | 87824.00 | 1.54 |
|  | 2012 | 128335.00 | 1.66 | 90422.00 | 2.30 | 37913.00 | 1.00 | 40191.00 | 1.31 | 88144.00 | 1.90 | 49066.00 | 1.63 | 46668.00 | 1.72 | 32601.00 | 1.62 |
|  | 2011 | 130286.00 | 1.68 | 91367.00 | 2.32 | 38919.00 | 1.02 | 40720.00 | 1.32 | 89566.00 | 1.92 | 49280.00 | 1.63 | 48468.00 | 1.79 | 32538.00 | 1.61 |
|  | 2010 | 137665.00 | 1.75 | 97013.00 | 2.41 | 40652.00 | 1.05 | 45187.00 | 1.52 | 92478.00 | 1.89 | 52110.00 | 1.69 | 48510.00 | 1.82 | 37045.00 | 1.74 |
|  | 2009 | 135878.00 | 1.81 | 94319.00 | 2.46 | 41559.00 | 1.13 | 42726.00 | 1.60 | 93152.00 | 1.93 | 52548.00 | 1.83 | 47674.00 | 1.84 | 35656.00 | 1.75 |
|  | 2008 | 135287.00 | 1.83 | 95872.00 | 2.54 | 39415.00 | 1.09 | 39958.00 | 1.54 | 95329.00 | 1.98 | 52333.00 | 1.85 | 46174.00 | 1.82 | 36780.00 | 1.81 |
|  |  | PYLL | PYLLR (‰) | PYLL | PYLLR (‰) | PYLL | PYLLR (‰) | PYLL | PYLLR (‰) | PYLL | PYLLR (‰) | PYLL | PYLLR (‰) | PYLL | PYLLR (‰) | PYLL | PYLLR (‰) |
|  | Index | Total |  | Male |  | Female |  | Urban areas |  | Rural areas |  | Eastern China |  | Central China |  | Western China |  |

PYLL, Potential years of life lost; PYLLR, Potential years of life lost rate; AAPC, Average annual percent change; CI, Confidence interval
